# Supplementary material for: TaGra: an open Python package for easily generating graphs from data tables through manifold learning
Source: PeerJ Comput Sci. 2025 Jul 25;11:e2986. doi: 10.7717/peerj-cs.2986 (PMC12453856; doi:10.7717/peerj-cs.2986)
Supplement: Supplemental Information 1 [file peerj-cs-11-2986-s001.pdf]

# SUPPLEMENTARY INFORMATION

## Another Diabetes type one dataset

The analysis of Takashi's Diabetes type one dataset (Takashi et al., 2019; Cerono and Chicco, 2024), with 67 rows and 20 features, reveals several limitations in utilizing graph representations for class separation and clustering, particularly for the target variable (insulin regimen). In Figure 1 and Table 1 , we present the outputs of the graph analysis given we have implemented the similarity method to generate the output graph.

The heatmap analysis shows substantial overlap between the two target classes (0: no insulin, 1: insulin regimen). Instead of clear diagonal dominance, the heatmap exhibits high off-diagonal values, indicating weak neighbor-based separability. This suggests that the similarity or distance metrics used in graph construction may fail to capture the distinguishing characteristics of the dataset's features effectively.

The degree distribution plot lacks the heavy-tailed structure typically observed in datasets with hierarchical connectivity. This limitation is likely due to the small sample size, which restricts the formation of significant hubs or peripheral nodes, reducing the graph's ability to represent hierarchical relationships effectively.

The community histogram demonstrates poor clustering performance, with both target classes present within the same communities. This result highlights the challenges faced by the community detection algorithm in resolving distinct class-based groupings, undermining its utility for actionable insights into class separability.

These limitations likely stem from two sources: (i) the construction of the graph from tabular data, where the similarity metrics may inadequately represent the relationships between data points, and (ii) the scarcity of data, which limits the graph's structural richness. Future work will explore advanced methods for graph generation, such as the dynamic approaches proposed by Carneiro and Zhao (2018), to address these challenges and improve the robustness of graph-based analyses for small and complex datasets.

Looking at Table S1, we can observe that despite the limitations in graph representation for this small dataset, the homophily score (0.63) and its corresponding statistical significance (chi-square p-value of 0.00 and Z-score of 3.37) still indicate some level of class separation. This supports our broader conclusion that the homophily metrics can detect target variable separation even when other graph metrics and visualizations suggest poor separation. However, the relatively modest homophily score compared to our other datasets highlights that the effectiveness of graph-based analysis is significantly diminished when working with small datasets where feature-based separation is not pronounced. While statistically significant, the practical utility of such separation for meaningful clustering or classification tasks remains limited, as evidenced by the mixed communities shown in the visualization. This reinforces our observation that both data size and inherent class separability are crucial factors that determine the effectiveness of graph-based approaches for data analysis.

**Table 1. Diabetes type one EHRs dataset – Comparison of three different graph representation methods.** Quantitative metrics for each graph construction approach. Each metric is defined in Section Methods.

| Metric | Similarity |
|---|---|
| Graph Density | $6.69 \times 10^{-2}$ |
| Average Clustering Coefficient | 0.46 |
| Connected Components | 9 |
| Largest Component Size (%) | 82.1% |
| Assortativity Coefficient | 0.078 |
| Community Count | 10 |
| Modularity Score | 0.52 |
| Homophily Score | 0.63 |
| Chi-square $p$-value | 0.00 |
| Homophily Z-score | 3.37 |

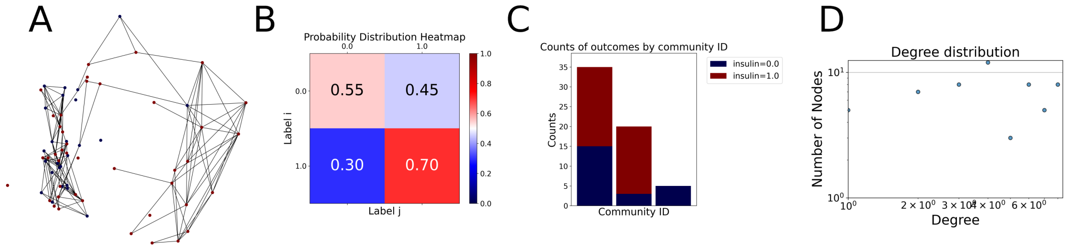


**Figure 1.** Outputs of the graph analysis module generated from the Takashi's diabetes EHR dataset (Takashi et al., 2019). (A) Graph visualization illustrates the connectivity patterns between data points based on similarity relationships, showing substantial overlap between the two target classes (red: insulin regimen, blue: no insulin). (B) Neighbor probability heatmap reveals poor class separation, with high off-diagonal values indicating weak neighbor-based clustering between the two target groups. (C) Community composition histogram shows mixed-label communities, indicating that the community detection algorithm fails to resolve clear groupings for the target variable. (D) Degree distribution plot lacks a typical heavy-tailed structure, suggesting insufficient hierarchical connectivity due to the dataset's small size. EHR: electronic health records.

# REFERENCES

Carneiro, M. G. and Zhao, L. (2018). Analysis of graph construction methods in supervised data classification. In *Proceedings of BRACIS 2018 – the 7th Brazilian Conference on Intelligent Systems*, pages 390–395. IEEE.

Cerono, G. and Chicco, D. (2024). Ensemble machine learning reveals key features for diabetes duration from electronic health records. *PeerJ Computer Science*, 10:e1896.

Takashi, Y., Ishizu, M., Mori, H., Miyashita, K., Sakamoto, F., Katakami, N., Matsuoka, T.-a., Yasuda, T., Hashida, S., Matsuhisa, M., and Kuroda, A. (2019). Circulating osteocalcin as a bone-derived hormone is inversely correlated with body fat in patients with type 1 diabetes. *PLOS One*, 14(5):1–11.
