# Supplementary figures and images for: TaGra: an open Python package for easily generating graphs from data tables through manifold learning
Source: PeerJ Comput Sci. 2025 Jul 25;11:e2986. doi: 10.7717/peerj-cs.2986 (PMC12453856; doi:10.7717/peerj-cs.2986)

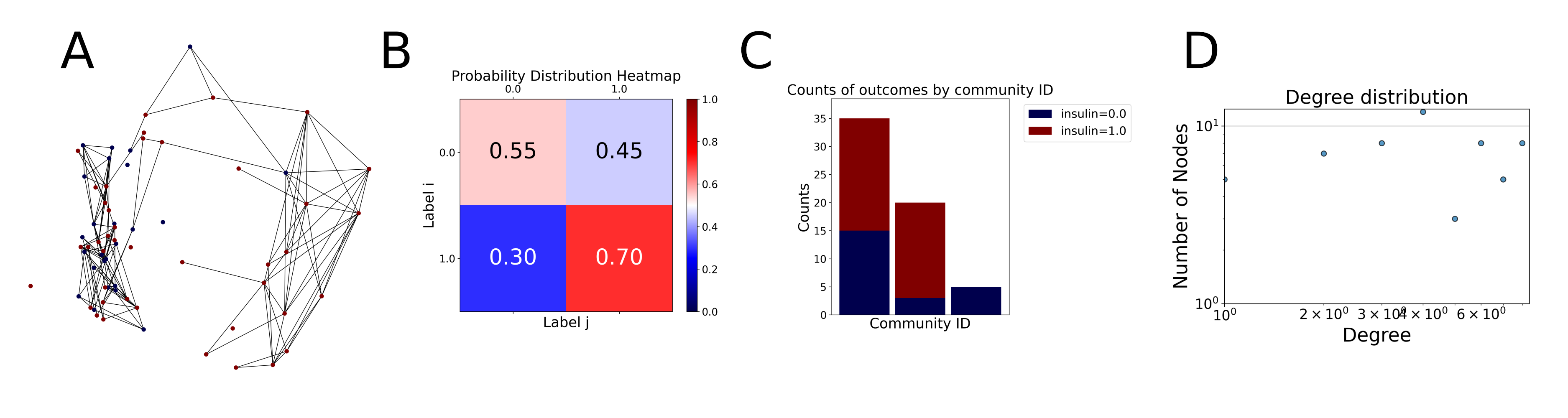

Supplement: Supplemental Information 2 [file peerj-cs-11-2986-s002.png]
